# Ants in a Labyrinth: A Statistical Mechanics Approach to the Division of Labour

**DOI:** 10.1371/journal.pone.0018416

**Published:** 2011-04-25

**Authors:** Thomas Owen Richardson, Kim Christensen, Nigel Rigby Franks, Henrik Jeldtoft Jensen, Ana Blagovestova Sendova-Franks

**Affiliations:** 1 Department of Engineering, Design and Mathematics, University of the West of England, Bristol, United Kingdom; 2 School of Biological Sciences, University of Bristol, Bristol, United Kingdom; 3 Institute for Mathematical Sciences, Imperial College London, London, United Kingdom; 4 Department of Physics, Imperial College London, London, United Kingdom; 5 Department of Mathematics, Imperial College London, London, United Kingdom; Universita' del Piemonte Orientale, Italy

## Abstract

Division of labour (DoL) is a fundamental organisational principle in human societies, within virtual and robotic swarms and at all levels of biological organisation. DoL reaches a pinnacle in the insect societies where the most widely used model is based on variation in response thresholds among individuals, and the assumption that individuals and stimuli are well-mixed. Here, we present a spatially explicit model of DoL. Our model is inspired by Pierre de Gennes' 'Ant in a Labyrinth' which laid the foundations of an entire new field in statistical mechanics. We demonstrate the emergence, even in a simplified one-dimensional model, of a spatial patterning of individuals and a right-skewed activity distribution, both of which are characteristics of division of labour in animal societies. We then show using a two-dimensional model that the work done by an individual within an activity bout is a sigmoidal function of its response threshold. Furthermore, there is an inverse relationship between the overall stimulus level and the skewness of the activity distribution. Therefore, the difference in the amount of work done by two individuals with different thresholds increases as the overall stimulus level decreases. Indeed, spatial fluctuations of task stimuli are minimised at these low stimulus levels. Hence, the more unequally labour is divided amongst individuals, the greater the ability of the colony to maintain homeostasis. Finally, we show that the non-random spatial distribution of individuals within biological and social systems could be caused by indirect (stigmergic) interactions, rather than direct agent-to-agent interactions. Our model links the principle of DoL with principles in the statistical mechanics and provides testable hypotheses for future experiments.

## Introduction

Both human and animal societies display a division of labour, in which there may be an unequal distribution of effort between or within particular tasks, according to age or experience [1], [2], sex [3], physiology [4] or morphology [5]. Such specialisation has long been known to improve collective productivity [6] because learning allows individuals that focus on a subset of tasks to perform more efficiently than generalists (note however the exception to the rule provided by Dornhaus, 2008). Division of labour is most advanced in the societies of insects such as ants, bees, wasps and termites [7]. Within an insect society, there is typically considerable individual variation in the sensitivity to stimuli associated with particular tasks. One of the simplest models of Division of Labour (DoL), the fixed-threshold model (FTM), invokes this individual variation in sensitivity to such task-related stimuli [8], [9]. There is good evidence for the existence of such response thresholds in ants [10], [11], [12], bumblebees [13], the honey bee [14], [15], [16], wasps [17] and termites [18]. Experiments also provide strong support for the role of response thresholds for the maintenance of colony homeostasis [13], [19], [20]. Individual variation in thresholds has genetic [21], [22], morphological [11], hormonal [23] and developmental [24] components. Although direct evidence for a positive relationship between colony fitness and wide threshold distributions is lacking, there is evidence in the honey bee that genetic variation (the number of patrilines within the colony) positively influences colony fitness [25].

In the FTM, the decision of an individual whether or not to undertake a particular task, such as foraging or brood care, is determined by two parameters; the sensitivity of the individual to stimuli associated with the task (its response threshold), and the level of demand for that task (the stimulus value). When an individual senses that the stimulus exceeds its threshold value, it becomes activated, and performs some work. Through such activity, sensitive (low threshold) individuals reduce the stimulus level such that it often does not reach the threshold of their less sensitive nestmates. This negative feedback loop homeostatically maintains the stimulus level (the task demand) at a steady state, around which it fluctuates. A further consequence of this mechanism, and one that matches the pattern observed in nature, is that the activity distribution becomes right-skewed; a small minority of sensitive individuals perform the majority of the work [13], [26], [27], [28], [29].

Here, we extend the FTM by explicitly including space. This modification induces a spatial 'percolation' effect [30], [31], [32] in which small differences amongst agents in their response thresholds, are related to large differences in their probabilities of performing work. The extension of the original fixed-threshold models of DoL to include space removes the assumptions that individuals and task-associated stimuli are *well-mixed.* The movement and activity of the individuals in the spatial fixed-threshold model (SFTM) may then be analysed as a case of diffusion in disordered media - a well studied branch of statistical mechanics [33], [34].

The FTM assumes that individuals and stimuli are well-mixed and that each individual experiences the same global stimulus level equally. This is a simplifying assumption. However, it is realistic only for a minority of cases when the stimulus is spatially uniform. For example, honey bees homeostatically maintain the nest temperature and CO_2_ levels within certain acceptable ranges [13], [20]. When it gets too hot inside the nest, the bees with the lowest threshold to temperature begin to fan their wings, thereby increasing the airflow and reducing the temperature such that it never reaches the thresholds of their less sensitive nestmates. So because temperature and CO_2_ levels can be expected to be fairly uniform within the nest, the assumption of perfect mixing of stimulus and bees is justified. Therefore modelling this process as a non-spatial process is reasonable. However, when the stimulus in question is heterogeneous over space, perfect mixing can no longer be assumed. To appreciate the importance of modelling DoL without the assumption of perfect mixing, consider the honey bee comb, organised- or rather compartmentalised- into different zones in which the cells contain either brood, pollen or honey [35]. Therefore, tasks are not uniformly distributed in space [36]. Furthermore, individuals themselves are not well mixed. Despite their high potential mobility, individual ants [37], honey bees [38], bumble bees [39] and wasps [40] tend to be faithful to particular parts of the nest and this spatial fidelity persists even when many tasks are removed [41].

In the FTM, the *distribution* of the individual response thresholds within the colony- the Colony Threshold Distribution (CTD)- will bear directly upon the proportion of individuals that are mobilised to respond to a given stimulus level. Indeed, the precise form of the CTD will have significant adaptive consequences [42]. Abrupt discontinuities in the CTD would affect the ability of the colony to produce an appropriate response to small changes in the demand for labour. For example, consider the scenario in which the colony is evenly split between two types of individual; half of the ants have low thresholds and the other half have high thresholds. In that case, the colony will be unable to produce a graded response to fluctuating stimulus levels, because only 0% (both thresholds above stimulus level), 50% (stimulus level above the low threshold but below the high threshold level) or 100% (both thresholds below stimulus level) of the individuals may be active at any one time. If, on the other hand, the CTD has a continuous distribution, the colony will produce a more finely graded response that is proportionate to the stimulus levels.

Experimental data on the form of the CTD is rather limited. To our knowledge, only in the honey bee, *Apis melifera* is there a quantitative description of the CTD, which is approximately Gaussian [20]. Indeed, several previous simulation studies of the FTM have assumed a Gaussian distributed CTD [19], [43]. For simplicity, we first consider the case of the uniform CTD. Uniform distributions lack any central tendency (they are not humped) and so have a variance, defined by the range of the distribution.

For completeness we also investigate the influence of a Gaussian CTD upon the ability of the colony to minimise both the total task demand and the spatial variation thereof. As well as the aforementioned uniform and Gaussian CTDs, we also explore the consequences of completely removing individual threshold variation.

## Methods

Before constructing a biologically relevant individual based model in two dimensions, we will introduce several important concepts and issues using the more abstract but simpler one-dimensional model [44], [45].

### i. A model of division of labour in one dimension

Let us imagine a colony of heterogeneous and mobile ants, along with their (stationary) brood, inhabiting a ring-shaped nest in which the ring cross-section is so narrow that ants may not pass one another, although they may pass over the brood. The brood are regularly spaced, so the distance separating brood items is fixed. This scenario is modelled using a one-dimensional cellular automata with 500 grid squares with periodic boundary conditions. Each grid square contains a brood item, and within a single time-step an ant may only move one grid-square, that is, from one brood item to an adjacent brood item. However, there are more brood items than adult ants, so unlike the brood, the ants need not be regularly spaced. Each brood item demands regular labour, for example, grooming and feeding. Let us further assume that the demand of the brood for attention is a stimulus that can be detected by nearby ants. To reflect this, the stimulus grows over time, a single brood item is selected every time-step and its stimulus is increased by a fixed amount - this is the stimulus ‘drive’. Given the ring-shaped nest geometry, and the fact that ants are unable to pass one another, it is reasonable to allow each ant to perceive only local information about its stimulus environment. Thus, each ant may only detect the stimulus level of the brood items within a ‘domain of care’. This domain of care is defined as the brood item that the ant is standing on, plus the two items immediately adjacent to the current item. When an individual ant detects that one of the brood items in its domain of care requires attention (i.e., that the brood item has a stimulus value greater than the threshold of the attending ant), the ant moves to the site and performs work on the brood item. The reduction in the demand for work is reflected by reducing the stimulus value to the threshold level of the attending ant.

There are several possible formulations of response threshold functions in which the probability of response is not binary, that is, not purely deterministic [8], [9], [46], [47]. Although probabilistic response thresholds may be more biologically realistic, they require the choice of an extra parameter that controls the steepness of the sigmoid curve. Hence, we simplify the response thresholds to a step function; when the stimulus is less than the threshold, S<*θ*, the response probability is zero, and when S>*θ*, the response probability is equal to one.

It has been shown that collective regulation of task demand is improved when the threshold distribution is broad [48], [49]. Following Page & Mitchell (1998) and Theraulaz (1998) inter-individual threshold variation was generated in the simplest manner possible; the thresholds, *θ*, were drawn from a uniform distribution 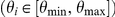 so sensitive ants were no more common than insensitive ants.

**Figure 1:**
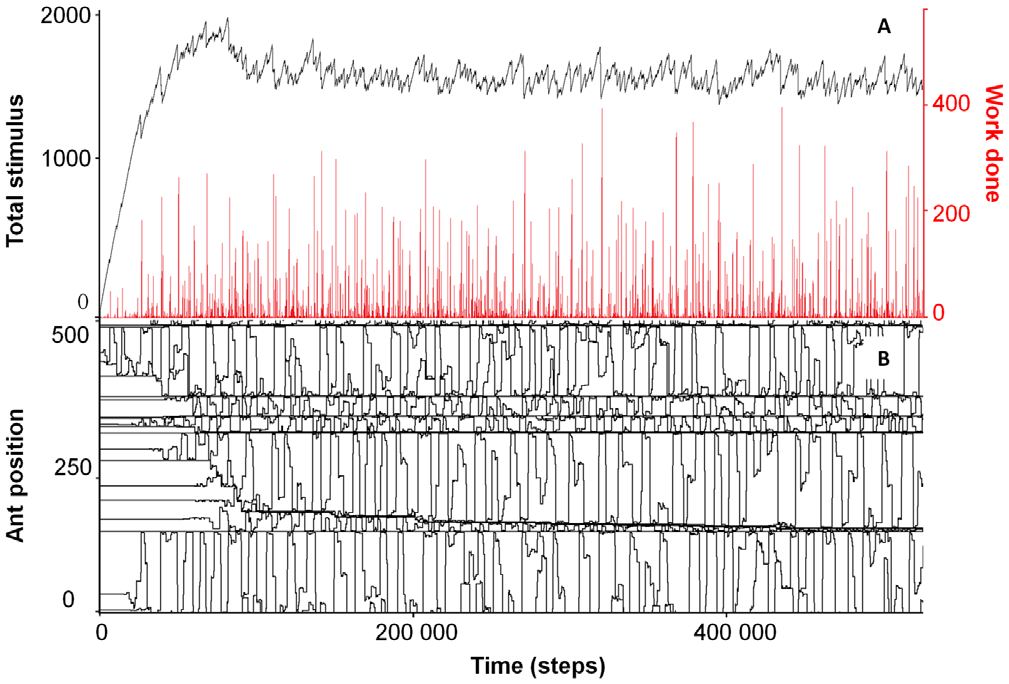
Emergence of one-dimensional spatial division of labour. a) Black line: The development of the steady state (world circumference = 500, N ants = 20, stimulus drive = 0.1 stimulus units per time-step). Red line: The total work done per time-step. b) The positions of the ants in the ring nest as a function of time. The ants measure their position clockwise from a fixed but arbitrarily chosen point along the ring. There is a transition from a random initial configuration, to one in which ants are aggregated into a few clusters, with low threshold ants shuttling between the clusters. The clusters are represented by the straight lines.

If the above scenario is initialised with a random spatial distribution of ants, and with all brood having zero stimulus, the total stimulus will grow at first, before reaching a steady state (Figure 1a). At the steady state, the increase of brood-care stimulus is balanced by the work performed by the ants. The collective output (the total work done per time-step) fluctuates intermittently.

The most interesting outcome of the one-dimensional model concerns the spatial distribution of the ants. Initially, the ants are distributed at random positions along the ring nest, however, as the model self-organises towards the steady-state the ants become aggregated into a few clusters. In Figure 1b at *t* = 0 the ants are randomly (uniformly) distributed around the ring, but when the simulation reaches the steady state at *t*∼50,000, five stable clusters form. Interestingly, the distance separating the clusters is fairly constant, that is, on average the clusters are regularly spaced (overdispersed).

**Figure 2:**
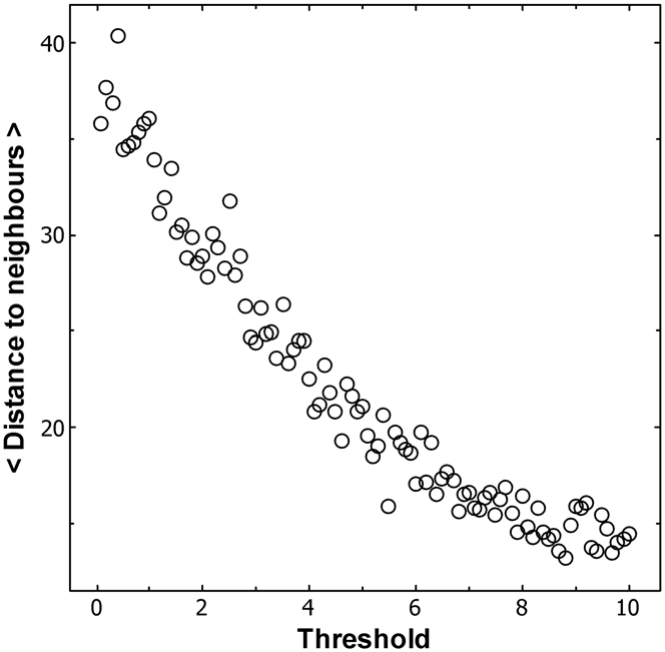
The mean distance to the left and right neighbours is an inverse function of the response threshold. Periodic boundary conditions apply. World circumference = 500, N ants = 20, stimulus drive = 0.1 stimulus units per time-step, N simulations = 500.

The mechanism behind this pattern is revealed when the average distance from every ant to its two neighbouring ants is plotted as a function of its response threshold (Figure 2). The greater the sensitivity of an ant (i.e. the lower its response threshold), the greater the distance separating the ant from its neighbouring ants. The clusters consist of ants with relatively high thresholds, while ants with relatively low thresholds shuttle between these clusters.

**Figure 3:**
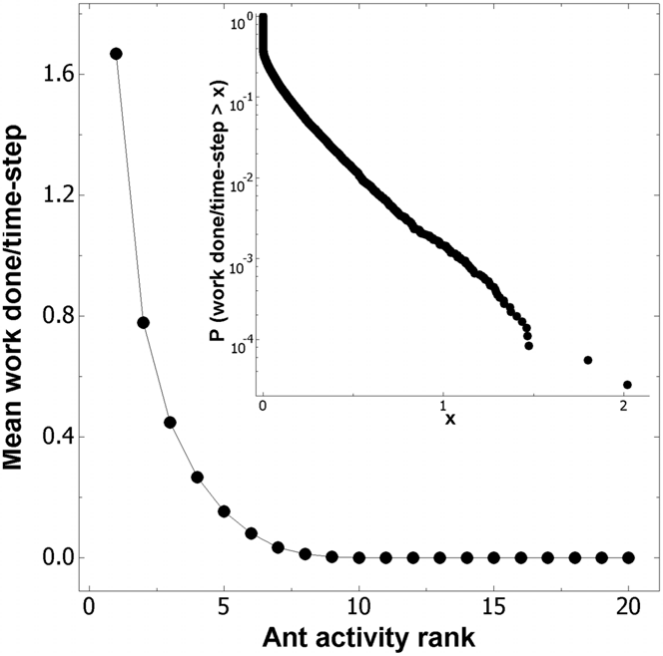
The skewed activity distribution. Individual ant activity is measured on a per-ant basis, as the work done per time-step. Main panel; an activity-rank plot. A rank of 1^st^ indicates the ant was the most active, and a rank of 20^th^ indicates the ant was the least active. Panel insert: the same data as the main panel plotted as the survivorship of the individual ant activity. The distribution is exponential-like. Model parameters as in Figure 2 legend. All realisations were run for 50000 time-steps after reaching the steady-state.

The observation that sensitive ants ‘box in’ their less sensitive nestmates, implies that the activity distribution is skewed. Indeed, when the total work done by each of the ants within a given period is accumulated, and divided by the time elapsed, one arrives at a useful measure of the individual activity; the work done per time-step per ant. As in real social insect colonies, the individual activity distribution is highly right-skewed so that a minority of the ants perform the majority of the work (Figure 3). As there are many alternative functions for plotting distributions [50], [51], for ease of comparison, Figure 3 shows the skewed activity distribution plotted using two common methods; a Zipf-type rank distribution [27], and a survivorship (the complement of the cumulative distribution) function [26].

In summary, when considering the task of attending brood items situated on each grid square in a one-dimensional nest, the activity of ants with a uniform distribution of threshold values and a finite domain of care is highly skewed. Relatively few ants with low thresholds will attend a majority of brood items while ants with higher thresholds cluster into groups and attend only a small fraction of the brood items. This skewness is induced by the spatial aspect and the indirect interactions among ants.

### ii. A model of division of labour in two dimensions

#### Pierre de Gennes' Ant in a Labyrinth

We will introduce the more biologically realistic two-dimensional model with a brief discussion of the similarity between the concept of a spatially explicit DoL in social insects and the classic ‘Ant in a Labyrinth’ model [32]. The importance of de Gennes’ model cannot be overstated; it laid the foundation for an entire new field in statistical physics, known as ‘diffusion in disordered media’. We will use the statistical tools of this approach [30], [52] to demonstrate the influence the inclusion of space has on the DoL when the 'disordered medium' is a collective environment, such as a social insect nest, filled with stimuli that vary in intensity across space. The aim of this approach is to explore the consequences of removing the assumption of well-mixed stimuli and agents.

In de Gennes' (1976) model, each site on a two-dimensional square lattice is occupied with probability p and hence unoccupied with probability 1-p. The probability p is known as the probability of occupancy. For example, if the occupation probability is *p* = 0.5, then, on average, half of the lattice sites will be occupied, and half unoccupied. A single ‘blind ant’ is then randomly dropped onto an occupied site on the lattice. The ant follows a simple rule: choose randomly one of the four adjacent (nearest-neighbour) sites (NESW). If the chosen site is occupied, the ant moves onto it, however, if it is empty, the ant does not move. Either way, the time is incremented by one unit. The ant is termed ‘blind’ because the initial four-way choice is made irrespective of those sites' occupancies.

A cluster is defined as a set of occupied sites which are connected by one of their four nearest neighbours such that any two sites in a cluster may be reached by a series of consecutive steps to the North, East, South or West. The mean cluster size, <*S*>, is the average cluster size to which an occupied site belongs (excluding the infinite percolating cluster, see later). If *p* is increased from *p* = 0, the mean-cluster size incresases and a critical point is reached (*p_c_ = 0.59274621...*) where the largest cluster spans the lattice, and the average cluster size, <*S*> diverges (goes to ∞). The lattice is then said to ‘percolate’. When *p*<*p_ c,_* clusters are finite, and then the ants are unable to percolate through the lattice even when given an infinite amount of time to do so. Such ants remain ‘trapped’ forever. When *p* = 1, all sites are occupied and there are no barriers to movement, so movement is Brownian. However, if the occupation probability is set to *p_c_* the ant displays ‘anomalous diffusion’, characterised by sub-diffusive movement [30].

#### A spatial fixed-threshold model of division of labour (SFTM)

There is only a single ant in the Ant in a Labyrinth model, the structure of the disordered medium - the labyrinth - is fixed and hence there are no ant-ant or ant-medium interactions [30]. The aim of de Gennes' (1976) model was to investigate diffusion in disordered media, and neither the term ‘ant’ nor the movement rules ascribed to the ant were intended to have a biological meaning. However, the model provides a framework upon which to extend previous non-spatial fixed-threshold models of DoL. In our spatial fixed-threshold model,the medium is treated as a landscape across which *many* ants move, and upon which they perform work, so altering its structure. The active ants thereby exert indirect influence on the activity of their nestmates. When inter-individual interactions operate indirectly through the medium of the shared environment, the process is a stigmergic one [53], [54], [55]. In the spatial fixed-threshold model (SFTM) both the movement and work rules assigned to the ants are derived from an earlier non-spatial fixed-threshold model of DoL [9].

The Ant in a Labyrinth model shows that anomalous diffusion occurs in a *static* disordered medium manually placed at a critical point (*p_c_*), however, anomalous diffusion may also occur in dynamical systems that self-organise to a critical point [56] without any external fine tuning of control parameters. In many models of self-organised criticality [52], [57], [58], ‘strain’ is slowly increased and is then released intermittently in spasmodic ‘quakes’. So the total amount of strain within the system increases until a steady state is reached where the slow ratcheting of the strain is, on average, balanced by intermittent dissipation of strain - termed ‘stick-slip’ behaviour. Similarly, in earlier non-spatial models of DoL, the competition between the demand for and performance of work is represented by adding a fixed amount of stimulus every time-step [9].

Our spatial fixed-threshold model is initialised on an empty lattice (all sites have zero stimulus, *S = *0), with periodic boundary conditions, across which a fixed number of ants were randomly distributed. A spatially random stimulus *δS* ‘rains’ onto the lattice such that in each time-step a single site is randomly chosen, and the stimulus value of the site is increased by a fixed amount, *δS*. This input is termed the stimulus drive.

The addition of even a single degree of freedom represented by the spatial domain, incurs a disproportionate increase in the complexity of the analysis of the individual and collective behaviour. Therefore for simplicity, binary response thresholds were implemented, rather than sigmoid or exponential response-probability functions [8]. So when the local stimulus exceeds the response threshold, the individual is activated with probability of one.

Many models of stick-slip behaviour in non-biological systems employ rules in which the strain is increased *only* when all sites are stable (inactive) and hence the system is in a quiescent state [59], [60], [61]. If this rule were imposed, the maximum number of active ants at any one time would be limited to one. In reality, the arrival of extrinsic task-related stimuli should be independent of the number of active individuals. Therefore a more biologically realistic scenario was implemented, in which stimulus input occurs every time-step irrespective of the activity status of the ants, thereby enabling many ants to work simultaneously [62]. In individual-based modelling, true concurrency is difficult to achieve. Individual concurrency was simulated in the following manner. Within each time-step, those individuals that detect an adjacent site containing a greater stimulus level than their threshold take turns to move and perform work. The turn-order is random, so individuals that find that an adjacent site exceeds their threshold by a very large amount *are not necessarily* selected to move before those individuals for which the adjacent site only contains marginally more stimulus than their threshold. Similarly, the turn-order is randomised from time-step to time-step, so if it happened that an individual was chosen to move first in the one time-step, this is not related to its turn-order in subsequent steps.

For simplicity, the majority of the analysis is based on a uniform colony thresdhold distribution (CTD), 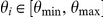, from which the individual thresholds were randomly assigned. For a uniform distribution, the variation is specified only by the width of the distribution which was fixed 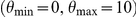, hence for the uniform CTD the threshold variation was constant (standard deviation, 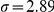). The effect of alternative CTD's was also investigated. The effect of a Gaussian CTD (with increasing standard deviation 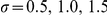, but with the same mean and range as the uniform CTD, 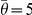, 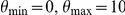) upon the ability of the colony to minimise the total task demand (the mean stimulus per site) and the spatial variation (the relative between-site variation) of the task demand, was also tested.

**Figure 4:**
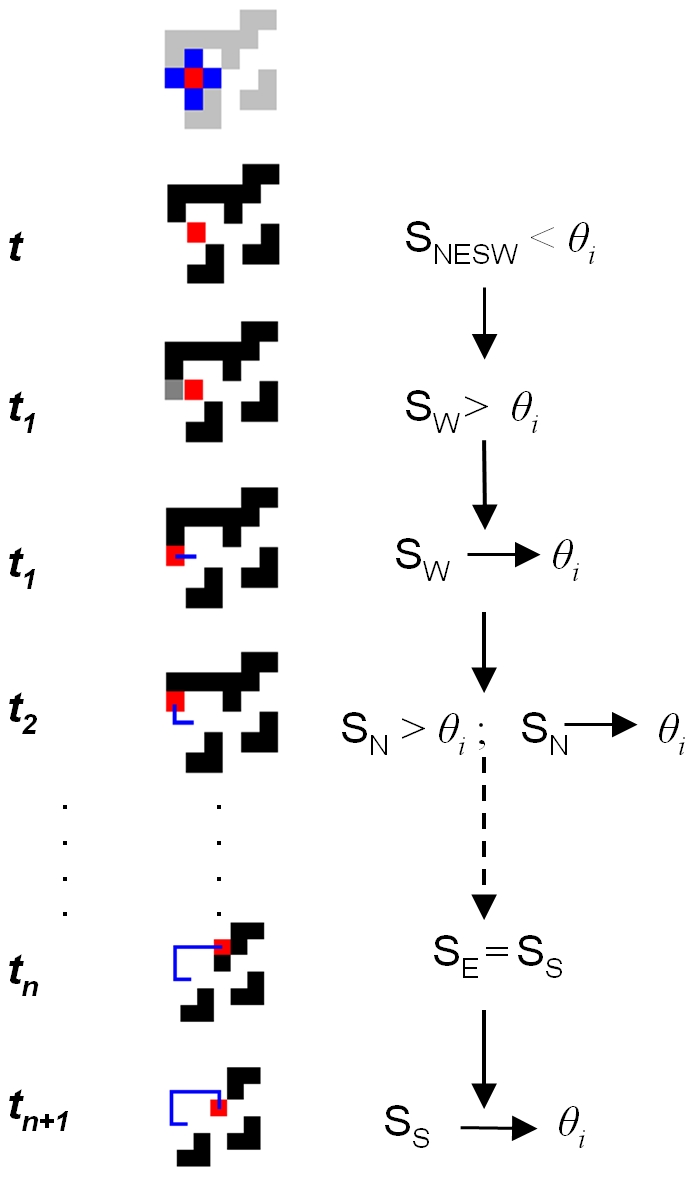
The stages involved in an 'ant bout'. The position of the ant is indicated by the red square. Each time-step every ant checks its local neighbourhood (the four blue squares) for any stimulus that exceeds its individual response threshold (S>*θ_i_*). Here, at *t*_1_ some stimulus arrives in the ant's West square, such that S_W_>*θ_i_*, so the ant moves onto it, instantaneously reducing the stimulus at that site to its threshold level, *θ_i_*. If more than one neighbouring site has S>*θ_i_*, the ant chooses randomly between them (at *t_n_*). At *t_n+1_* the ant has exhausted the stimulus in its four adjacent squares, so it is trapped.

**Figure 5:**
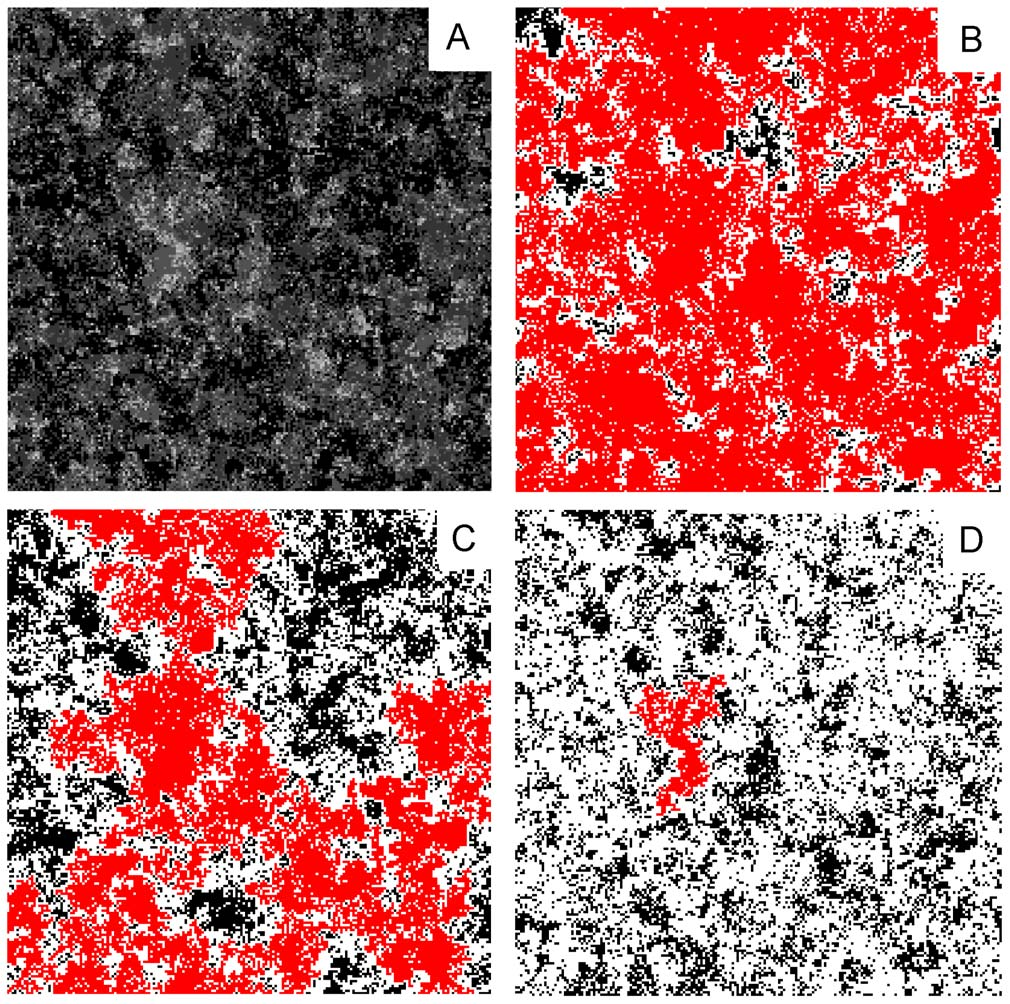
The stimulus landscape percolates at a critical response-threshold. a) A stimulus landscape as it appears to the outside observer (*δS* = 1, 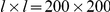, N ants/*l* ^2^ = 0.04). The more stimulus a site contains, the darker the grey. b) Threshold-dependent site-occupancy for the same landscape as seen by a sensitive ant (*θ_i_* = 1, *p* = 0.759). Sites with S<*θ_i_* are white. The largest cluster on the lattice is coloured in red. The cluster ‘percolates’ across the lattice. c) Threshold-dependent site-occupancy for an ant with *θ_i_* = 1.55, here *p* = 0.594. The occupancy is just above the critical occupancy (*p_c_ = *0.5927…), where the mean cluster area displays a phase-transition. d) Threshold-dependent site-occupancy for a less sensitive ant, where 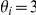 and *p* = 0.32. To this ant most sites do not contain stimulus, clusters of occupied sites do not span the lattice, and hence the landscape does not percolate.

To an outside observer, the stimulus landscape structure is viewed as a surface that varies continuously across time and space (Figure 4, Figure 5a). Sites may contain any stimulus value in the range 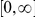. However, to the ant *i*, the labyrinth is viewed through the binary lens of its threshold; each site either contains a detectable amount of stimulus (S>θ*_i_*) or it does not (S<θ*_i_*). We define a site to be occupied (from a given ant's perspective) if the amount of stimulus in a site if greater than the threshold of the ant.

**Figure 6:**
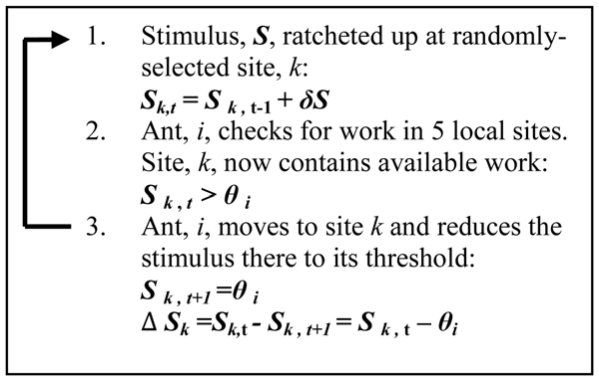
Stimulus input and ant activity update rules for the two-dimensional model.

If an ant detects that either its current site or one of the four nearest neighbour sites (NESW) bordering the current site is occupied (i.e., that any of those five sites has *S*>*θ_i_*) it moves to do work there, but otherwise remains inactive. If more than one neighbouring site is occupied (has S>θ*_i_*) the ant makes a random choice. After moving to a site, the ant then performs some labour there, and reduces the stimulus to its threshold level, *S* = *θ_i_* (see Figure 6).

So upon encountering a site containing a given stimulus value, a low threshold ant *i* will reduce the stimulus there to a lower level (i.e., it will do more work) than a high threshold nestmate *j*, because θ*_i_*<θ*_j_*. Thus unlike previous models [8], [9], [47], the amount of work done by an ant in a time-step (its efficiency) is a function solely of the ants' threshold and its location. This avoids the need for additional assumptions, for example, having to specify the stimulus reduction (task performance efficiency) as a function of the number of active individuals working [8], [47]. Our linking of efficiency to the threshold is reasonable; in the honey bee, corpse-removal efficiency is positively correlated with the degree of specialisation upon the task, and thus presumably also with the sensitivity to the stimuli associated with honey bee corpses [63].

In summary, the SFTM builds upon de Gennes' (1976) model by including ant-medium interactions which, through stigmergic modification [53], [54], [55], [64] of the medium, generates ant-ant interactions that are indirect but still causal. For example, the action of a sensitive ant working in the cell adjacent to a less sensitive ant will reduce the probability that the less sensitive ant is active in the next time step.

### iii. Rationale for model analysis

#### Stimulus landscape structure and threshold effect on activity

**Figure 7:**
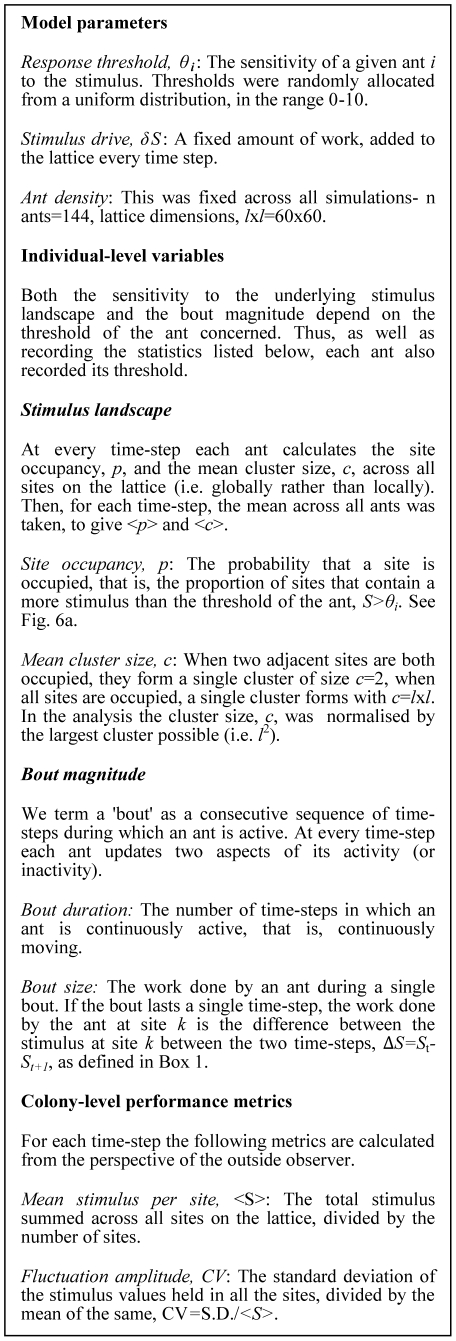
Definition of parameters and response statistics for the two-dimensional model.

The two-dimensional model is based on interactions between the stimulus landscape and the activity of individual ants. However, the variation in the sensitivity amongst the ants means that the 'perception' (i.e. *local* detection) of the stimulus landscape is dependent upon the threshold of the ant concerned (Fig. 5). Therefore those measurements concerning the landscape structure (site occupancy and cluster size) were calculated for each ant and then averaged across all ants (Figure 7). Because the model was run at various different driving rates, *δS*, for ease of comparison, the measures of landscape structure were normalised by the driving rate.

The highly skewed distribution of activity [27] that characterises many insects may in some cases follow a power law [26]. Hence, we examined the cumulative probability distributions for the measures of landscape structure. This technique has a low margin of error in estimating the power-law exponent, *α*, of the probability density (*P*(*x*) ∼1/*x^α^*) [51].

We also examined how individual sensitivity to the stimulus influences how the ants 'perceive' the structure of the stimulus landscape and thus the amount of work they perform upon it. To do so we plotted both the mean cluster size, and the amount of work performed by an ant during a 'bout' of continuous activity (Figure 7), as a function of the response threshold.

#### Spatial homeostasis and the colony threshold distribution

The ability of a social insect colony to buffer its internal environment against the fluctuations of the external environment has direct fitness consequences for the colony [25], [49]. It should be beneficial for a social insect colony to minimise the absolute demand for the task (the total stimulus) as well as its spatial variation.

Our first performance metric is the mean stimulus per site, <S>, which quantifies the absolute demand for work per site; the greater <S> the more work is required (Figure 7). If we assume that it is maladaptive for a colony to allow the demand for a task (the stimulus) to rise unchecked, then the lower <S>, the better the colony performance.

While the above metric concerns the average demand for labour per site, the second performance metric, the fluctuation amplitude, measures the between-site variation of the stimulus (Figure 7). As the sites are distributed across space, the fluctuation amplitude measures the degree of spatial heterogeneity in the stimulus. This dimensionless number was used to make comparisons of the *relative* amount of variation across different drives. A similar measure of the relative variation in models of termite building [65], [66] and ant brood tending [67] have been previously implemented, although in those studies the measure was termed the ‘fluctuation amplitude’.

Why might it be beneficial to minimise the between-site fluctuation amplitude? Let us assume there are penalties when the stimulus held on a site exceeds a given value. For example, suppose the sites represent brood items each with an associated hunger stimulus. There should be a stimulus level which, if exceeded, will cause the brood item to die. Let us give a concrete example; suppose there are 100 brood items, and that a brood item will die if its hunger stimulus exceeds *S_i_* = 7. Now suppose that the ants can provide enough brood care to reduce the *total* stimulus to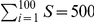, that is, <*S*> = 5. If the ants allocate their labour completely evenly (i.e. if there is perfect mixing), then every item will have *S* = 5. However, when the tasks and stimuli are imperfectly mixed, assuming an even distribution of labour across space may not be realistic. For example, workers of the bumble-bee *Bombus impatiens*, unevenly distribute their brood care across space, which may cause increased size-differences between the brood [68]. Suppose then, that this unequal allocation of effort results in a Gaussian distribution of the stimulus across space. If the ants manage to minimise the standard deviation of the stimulus distribution (<*S*> = 5, S.D. = 0.5) then the probability that a brood item dies is, 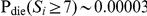. However, if the same amount of effort is more unevenly allocated, resulting in a doubling of the standard deviation (<*S*> = 5, S.D. = 1.0), the probability that a brood item dies is 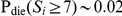; an increase of three orders of magnitude.

We compared the performance of the different CTDs against the null scenario in which the amount of stimulus held at a site is exponentially distributed. As any exponential distribution has CV = 1, a distribution with CV>1 has a higher variance in units of the mean value than the null expectation, whereas CV<1 indicates a lower variance than expected.

#### Ant-ant distances

The one-dimensional model indicated that non-random spatial distribution of individuals could emerge through threshold-based spatial mutual exclusion. Such patterns emerged purely as a result of *indirect* ant-ant interactions mediated via the stimulus landscape, rather than through explicit ant-ant interactions [69] such as attraction or repulsion. However, in the one-dimensional model the ants could not pass one another. In the two dimensional model, this spatial restriction is lifted.

To test for the presence of such non-random spatial distributions, the relationship between each ant and its nearest-neighbour ant was measured in terms of their respective thresholds and their separation distance. Thus, for each ant, three observables were recorded: the distance to the nearest neighbour ant, and the thresholds of the ant, *θ_i_*, and its nearest-neighbour, *θ_NN_*. From the latter two observables, the threshold *difference* was calculated by subtracting the threshold of the nearest neighbour ant from that of the active ant (*θ_i_*-*θ*_NN_). Thus if the active ant is more sensitive (has a lower threshold) than its nearest neighbour, as is most often the case, the threshold difference is negative, whereas if the nearest neighbour is more sensitive than the active ant, the threshold difference is positive.

## Results

### Stimulus landscape structure and threshold effect on activity

Low-threshold ants observe a landscape in which finite islands of unoccupied sites (those the ant perceives as containing no stimulus) are embedded within a percolating stimulus ‘sea’ (Figure 5 b,c). Conversely, insensitive ants observe a landscape in which stimulus islands do not span the lattice, hence for these ants the clusters holding available work do not percolate (Figure 5 d).

**Figure 8:**
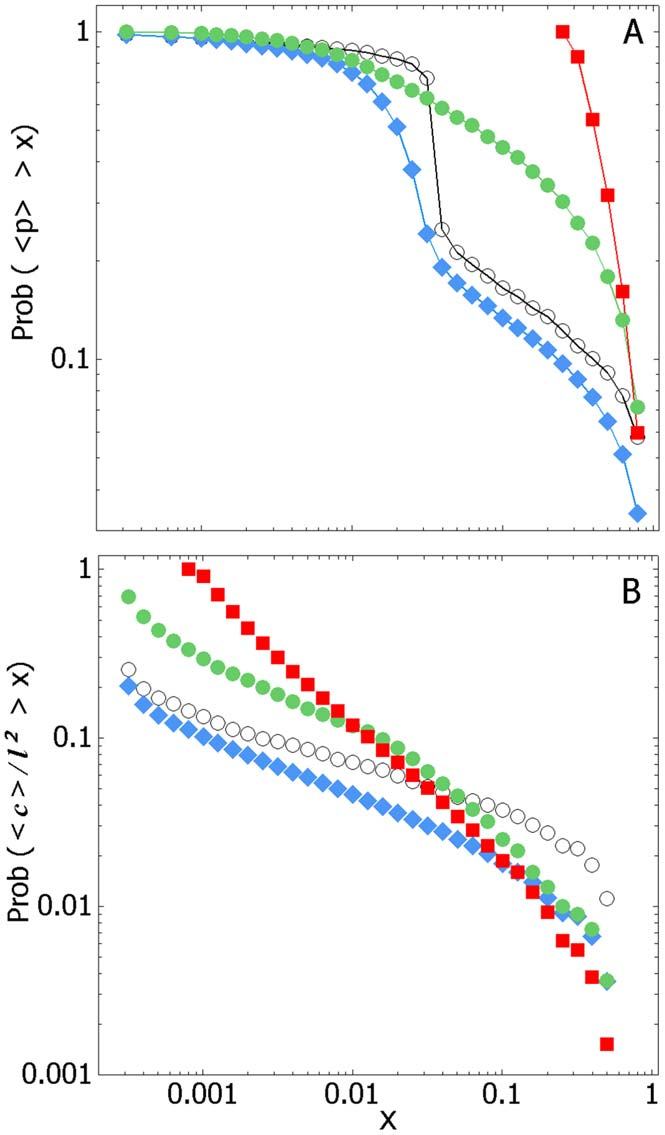
The scale-free structure of the stimulus landscape. Both panels depict the survivorship (the complement of the cumulative distribution) function for: a) mean site occupancy, <*p*> and b) The mean cluster size, <c>, normalised by the maximum cluster possible, *l*×*l*. Both <*p*> and <c> are ensemble-averages, calculated by averaging across all individuals irrespective of threshold. The different curves represent different fixed drives (○; δS = 1×10^−2^, ⧫; δS = 1×10^−1^, •; δS = 1×10^0^, ▪; δS = 1×10^1^).

The survivorship of the site occupancy, <p>, displays a discontinuity that is more exaggerated, the lower the stimulus drive, δS (Figure 8a). The mean cluster size, <c>, of occupied sites displays a power-law distribution, so the structure of the stimulus landscape appears scale-free, i.e. fractal (Figure 8b).

**Figure 9:**
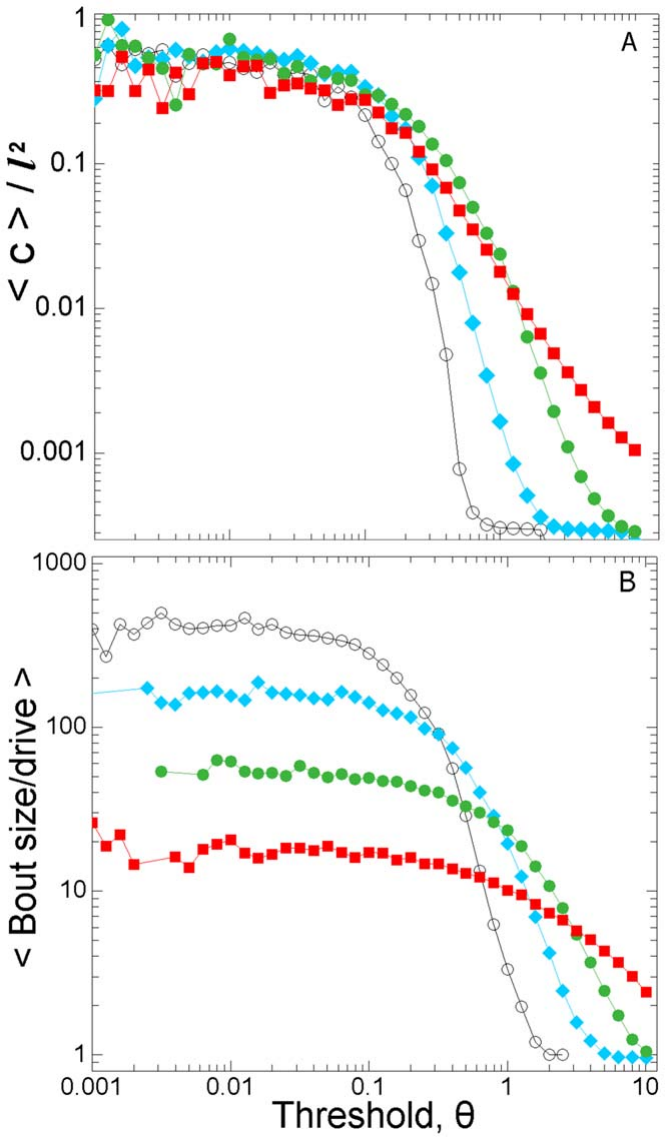
The structure of the stimulus landscape- and hence also the bout magnitude- are nonlinear functions of the individual response-threshold. **a**) Mean cluster size, <c>, normalised by dividing by the maximum cluster possible, *l* ^2^ and b) Mean standardised bout size (size/drive) for individual ant-bouts. Ants were assigned to threshold bins of logarithmically increasing width. The different curves represent different fixed drives (N simulations per drive = 500, ○; δS = 1×10^−2^, ⧫; δS = 1×10^−1^, •; δS = 1×10^0^, ▪; δS = 1×10^1^).

A small decrease in the threshold results in a disproportionate increase in the ant’s perception of the average cluster size, that is, the amount of work available (Figure 9a). This disproportionality is translated into a non-linear relationship between the threshold and the work done (Figure 9b). However, the standardised bout size is not a simple function of the threshold of the active ant, but also depends upon how fast the system is driven. That is, for a given threshold value, *θ_i_*, the lower the driving rate the greater the standardised bout size.

### Spatial homeostasis and the colony threshold distribution

**Figure 10:**
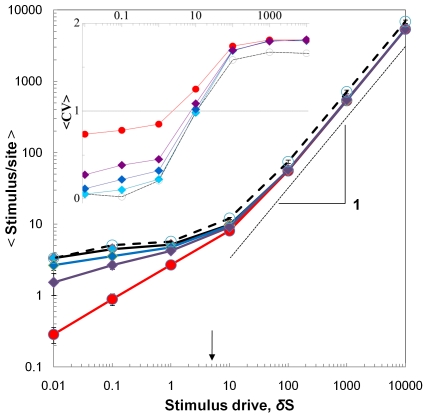
The mean stimulus per site as a function of the stimulus drive. The different symbol types represent different colony threshold distributions (•; Uniform CTD, minimum = 0, maximum = 10, ⧫; Gaussian CTD, S.D. = 1.5, ⧫; Gaussian, S.D. = 1.0, ⧫; Gaussian, S.D. = 0.5, ○; Homogeneous CTD (all ants are identical), θ = 5, *l*×*l* = 60×60, ant density = 0.04, N simulations per parameter combination = 60). The error bars are standard deviations. The thin dashed line has a slope of one. Insert: The fluctuation amplitude (CV = S.D./<S>) for the stimulus held across all sites on the lattice as a function of the drive. The horizontal line indicates the null expectation, that is, when the amount of stimuli held in a site is Poisson distributed.

We first examine the mean stimulus per site as a function of the stimulus drive. For all colony threshold distributions, the mean stimulus per site increases nonlinearly with the driving rate (Figure 10). When the amount of stimulus added to the lattice is large (*δS*>10), the mean stimulus per site is a linear function of the drive, hence the gradient of the stimulus per site in Figure 10 is ∼1 when *δS*>10. However, when the size of the stimulus input is low (*δS* <1), the mean stimulus per site increases as a sub-linear function of the drive. For example, for the uniform CTD, a tenfold increase in the drive from *δS* = 0.01 to *δS* = 0.1 only results in a threefold, but not a ten-fold, increase in the mean stimulus per site (S = 0.3 to S = 0.9).

For all stimulus drives, the greatest stimulus per site was always when the ants were identical (thick dashed black line, Figure 10). As the threshold variation increased (the Gaussian CTD's with increasing standard deviation), the amount of stimulus per site decreased (the diamond shaped points, Figure 10). When the ants' thresholds were drawn from the uniform CTD, they maintained the total stimulus at a lower level than both the Gaussian and homogeneous CTD's. Therefore the greater the central tendency of the CTD (the more humped it is), the more stimulus per site. If it is adaptive for a colony to minimise the stimulus per site, that is, the work available, it would be advantageous for the CTD to exhibit a large variation around the mean. So the greater the variation between individuals, the better able they are to minimise the level of task. The relative advantage would be greater at low stimulus drives.

The differences between the CTD's were greatest at low drives. For example, when *δS* = 0.01 the average stimulus per site for a Gaussian CTD with standard deviation S.D. = 1.0 (S = 2.7) was nine times greater than for a uniform CTD (S = 0.3), whereas when *δS* = 1000 the mean stimulus per site for that Gaussian CTD (S = 583) was only 1.09 times that of the uniform CTD (S = 536).

We now turn to the relative between-site variance of the stimulus, as measured by the fluctuation amplitude (i.e. the coefficient of variation). The greater the driving rate, the greater the relative spatial variation in the stimulus. More precisely, the coefficient of variation increases as a sigmoid function of the drive (Figure 10 insert). When *δS*≤1, the spatial variation is *lower* than that produced by the 'null' Poisson distribution across sites, whereas when *δS*≥10, the spatial variation is *greater* than that produced by a Poisson.

It is interesting to note the trade-off between the mean stimulus per site and the fluctuation amplitude. Ideally, a CTD should minimise both. However, the CTD with the greatest threshold variation- the uniform distribution- produced the lowest stimulus per site, but the greatest fluctuation amplitude (Figure 10). Conversely, the CTD with the least variation- the homogeneous distribution- resulted in the greatest stimulus per site, but the lowest fluctuation amplitude. In summary, the greater the threshold variation, the better able the colony is to minimise the total demand for work, but the greater the relative spatial fluctuation amplitude.

### Ant-ant distances

**Figure 11:**
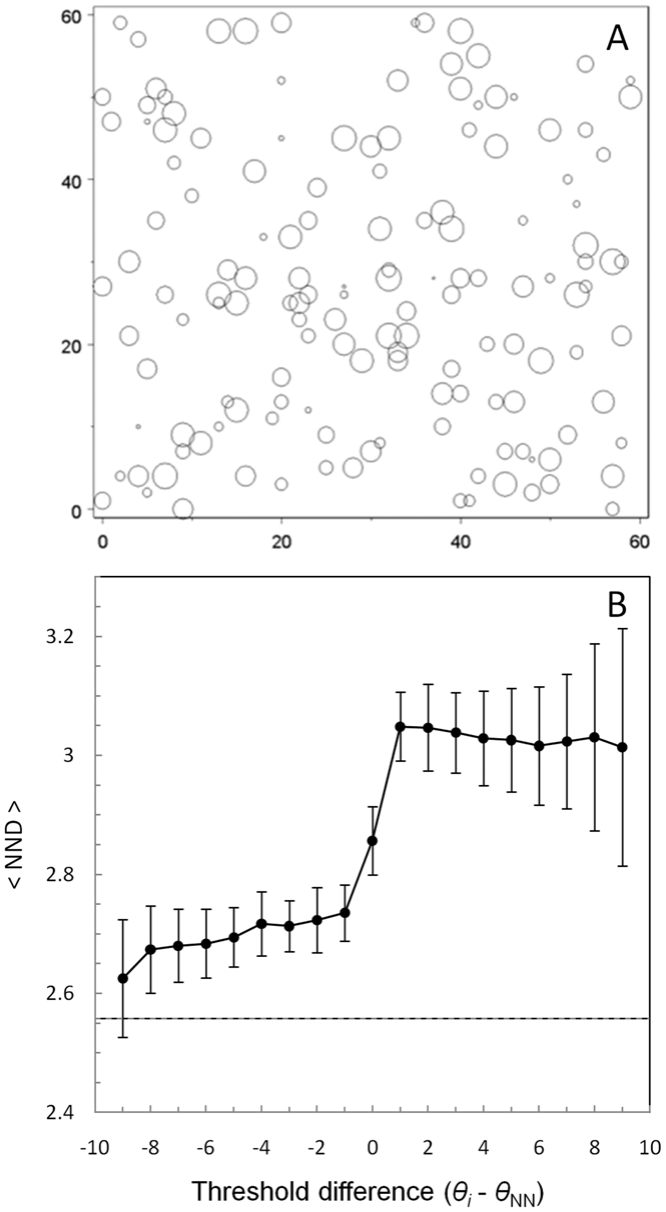
The distance separating neighbouring ants depends upon the difference in their response-thresholds. a) Map of ant locations. Symbol sizes are proportional to the threshold of the ant, so sensitive ants have small symbols. b) Mean distance between an active ant and its nearest neighbour (NND), as a function of the *difference* in sensitivity between the two (*θ_i_* - *θ*_NN_). Error bars are one standard deviation. Drive, *δ*S = 10, N simulations = 100, N time-steps per simulation = 5×10^4^. The horizontal line shows the expected NND under conditions of complete spatial randomness (Expected NND = 2.56, *σ* = 1.25, N simulations = 2000).

When an active ant has a lower threshold than its nearest neighbour ant, the distance separating the two tends to be small. Conversely, when the active ant has a higher threshold than its nearest neighbour ant, the separation distance tends to be relatively high (Figure 11). So insensitive individuals are indirectly ‘repelled’ by proximity to their more sensitive nestmates.

## Discussion

As in earlier non-spatial Division of Labour models based on thresholds, the SFTM reproduces the massively right-skewed activity distribution observed in real social insect colonies [13], [26], [27], [70], [71] as the vast majority of the labour is performed by a highly active minority (Figure 3).

In both the one- and two-dimensional models imperfect mixing of heterogeneous individuals and stimuli generated a non-random spatial structure of both the individuals (Figure 1b & Figure 11) and the stimuli (Figure 5, Figure 8 & Figure 10). Non-random distributions of individuals or task-associated stimuli are ubiquitous within social insect colonies. Complex spatial structuring of the distribution of individuals or task associated stimuli within social insect colonies includes the aggregation of individuals by role or caste [72], [73], [74], clustering of nest-building material [75], [76], [77] dead individuals [78] and brood [79], [80].

Whilst it is known that cells, individuals and societies can achieve some degree of homeostasis by minimising the temporal fluctuations of relevant stimuli [19], [24], [49], [81], it is important to emphasise that homeostasis may also be achieved by minimising these fluctuations across space. In many cases this capability will be highly adaptive. For example, it might be advantageous to minimise the spatial variation of stimuli associated with brood hunger, as the brood may die when a critical hunger is exceeded. In our model for all CTD's the spatial fluctuation amplitude of the stimulus is minimised when the stimulus drive is relatively low (i.e., when the drive is less than the average threshold, 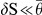, Figure 10), which is actually when the activity skew is greatest (9b). So spatial homeostasis is maximised when the division of labour is greatest.

Let us now turn to the issue of interactions and competition for work between individuals. Circumstances in which many individuals ‘graze’ a stimulus surface are ubiquitous in biology. For example, the removal of parasitic fungal species from the fungus gardens in fungus growing ants [82], brood sorting and tending [79], and the general activity of honey bee inside-nest workers [36] all involve multiple individuals moving across and performing work upon a spatially and temporally variable stimulus landscape. Clearly an individual performing work on the stimulus landscape causally influences the subsequent activity, or often the lack thereof, of its nestmates. On the rare occasions when a high threshold ant is active, it tends not to move very near its low threshold nestmates (Figure 11). This is because those nestmates have reduced the stimulus in the surrounding sites to such a low level that they appear to contain no work, so those sites act as barriers to movement. Conversely, when the active ant has a lower threshold, it is not ‘repelled’ by a higher-threshold neighbour ant, because that neighbour only reduced the stimulus in the area to its own threshold level, and no lower, hence the active ant detects that those sites contain work. Thus apparent aversion between behavioural or morphological castes [8], [10] resulting in spatial segregation of individuals might arise from indirect spatial exclusion rather than direct repulsion.

In ecology, explicit con-specific attraction or repulsion is often invoked to explain the observation of non-random spatial patterns, such as over or under-dispersion. The clustering of agents (under-dispersal) is associated with attraction, whereas regularly spaced agents (over-dispersal) is related to repulsion. In ants, spatial clustering of individuals has been explained by invoking *direct* inter-individual attraction and repulsion based on physical or behavioural differences between individuals [83], [84]. Similarly, spatial DoL through the segregation of physical castes has been explained by invoking explicit between-caste aversion [10]. At the colony level a high degree of regularity in the spacing of ant nests is ubiquitous and is conventionally understood in terms of competition for space [85], [86], [87]. One of the main results of this paper is that the spatial patterns previously ascribed to individuals that ‘pay attention’ to the proximity of their nestmates, can also be produced when individuals do not directly account for the proximity of nestmates. This conclusion is concordant with the concept of self-organisation through stigmergic processes [53].

Finally, we wish to highlight the scale-free structure of the stimulus landscape (Figure 5 & 6) and the similarity of the sigmoid threshold-activity functions (Figure 9) to phase-transition curves. Such phenomena are typical of complex systems at critical points [52], [88] and suggest that threshold-based DoL can self-organise towards a critical point.

Division of labour characterises all levels of biological organisation as well as human and artificial social systems. Our spatial fixed-threshold model links this organisational principle with the statistical mechanics approach to complex systems and provides testable hypotheses for future experiments.
